# Performance of conceptual framework elements for the retrieval of qualitative health literature: a case study

**DOI:** 10.5195/jmla.2021.1150

**Published:** 2021-07-01

**Authors:** Tove Faber Frandsen, Christina Louise Lindhardt, Mette Brandt Eriksen

**Affiliations:** 1 t.faber@sdu.dk, Professor WSR, University of Southern Denmark, Denmark; 2 clindhardt@health.sdu.dk, Department of Geriatric Medicine, Odense University Hospital, Odense Denmark. Department of Clinical Institute, University of Southern, Odense, Denmark; 3 mettee@bib.sdu.dk, University of Southern Denmark, Denmark

**Keywords:** systematic searching, qualitative reviews, PubMed, CINAHL, conceptualization models

## Abstract

**Objective:**

A growing volume of studies address methods for performing systematic reviews of qualitative studies. One such methodological aspect is the conceptual framework used to structure the review question and plan the search strategy for locating relevant studies. The purpose of this case study was to evaluate the retrieval potential of each element of conceptual frameworks in qualitative systematic reviews in the health sciences.

**Methods:**

The presence of elements from conceptual frameworks in publication titles, abstracts, and controlled vocabulary in CINAHL and PubMed was analyzed using a set of qualitative reviews and their included studies as a gold standard. Using a sample of 101 publications, we determined whether particular publications could be retrieved if a specific element from the conceptual framework was used in the search strategy.

**Results:**

We found that the relative recall of conceptual framework elements varied considerably, with higher recall for patient/population (99%) and research type (97%) and lower recall for intervention/phenomenon of interest (74%), outcome (79%), and context (61%).

**Conclusion:**

The use of patient/population and research type elements had high relative recall for qualitative studies. However, other elements should be used with great care due to lower relative recall.

## INTRODUCTION

A qualitative systematic review or qualitative evidence synthesis provides answers or gains a deeper understanding of the what, how, or why of a phenomenon [1]. Qualitative systematic reviews focus on summarizing, analyzing, and/or interpreting. The number of qualitative systematic reviews and evidence syntheses has increased dramatically during the last two decades, with many different approaches in existence [2, 3].

The increase in the number of qualitative studies within health care leads to a growing volume of studies addressing methods of systematically reviewing, integrating, and synthesizing findings from original qualitative studies. The Cochrane Qualitative and Implementation Methods Group is continuously involved in developing guidance on the synthesis of qualitative and mixed-method implementation evidence [4–8]. Recent studies addressing methods for systematically reviewing and synthesizing findings include those on the choice of database [9], choice of search strategy [10], and use of search filters to limit to qualitative literature [11, 12].

How to structure review questions has received a great deal of attention. Within clinical research, the population-intervention-comparison-outcome (PICO) model is often used to structure the review question, although other conceptual frameworks are available that are better suited for specific review types [13]. For qualitative systematic reviews, PICO may not be adequate [14], and thus models have been specifically developed for this review type [15–17]. A review from 2016 lists as many as twelve different models for structuring the qualitative review question and recommends that “in the absence of empirical data on effectiveness of structured approaches, the question structure should be selected to match the purpose and focus on the review” [3].

Two organizations are dedicated to strengthening conceptual frameworks and guidelines for conducting qualitative systematic reviews: Cochrane Collaboration and Joanna Briggs Institute (JBI), both of which are recognized as producers of systematic reviews that undergo rigorous scrutiny [18, 19]. Cochrane argues for the strength of simple question models but also recommends the use of a formulation that includes context [20] as stressed in earlier studies [21, 22]. Thus, Cochrane recommends the use of an extended model named PerSPEcTiF [20], which consists of the following elements: *perspective, setting, phenomenon of interest/problem, environment, comparison* (optional), *time/timing*, and *findings* [23]. JBI recommends population, phenomena of interest, and context (PICO) and argues that there is no need for an outcome statement in qualitative synthesis, as the expression of the phenomenon of interest represents the outcome [24].

A conceptual framework is also recommended for developing search strategies [25], although not all elements provide high recall [26]. The effect of the choice of model on search quality remains inconclusive [27]. One should keep in mind that when using a conceptual framework consisting of many elements (e.g., SPIDER or PerSPEcTiF), using all elements in the search strategy will, in practice, reduce recall [16]. Consequently, a search strategy should include only some elements, but little evidence is available to inform that process. Cochrane generally recommends omitting outcome from the search strategy, which is supported by a recent case study [26]. However, in systematic qualitative reviews, outcome is not necessarily included in the conceptual framework and therefore not necessarily relevant to consider for the search strategy. Furthermore, little is known about the retrieval potential of elements in conceptual frameworks available for systematic qualitative reviews.

Essentially, qualitative reviewers need to know how to locate relevant studies, which implies knowing which databases to search and how to search them. The present study contributes to this knowledge base by examining the impact of conceptual frameworks developed for qualitative reviews on retrieval. The purpose of this case study is to evaluate the retrieval potential of each element of conceptual frameworks in qualitative systematic reviews in health sciences.

## METHODS

To investigate the presence of elements from conceptual frameworks in publication titles, abstracts, and controlled vocabulary, we used a set of qualitative systematic reviews identified in a study of database coverage of qualitative health literature [9] as our starting point and analyzed whether the studies included in the reviews would be retrieved if a reviewer used the elements specified in the review to perform a thorough search for relevant studies. We defined a “thorough search for relevant studies” as one that uses synonyms as well as narrower terms as both text words and subject headings. Search terms were defined in this study using the thesauri available in PubMed and CINAHL and were found using entry terms in PubMed as well as narrower terms in both thesauri. We did not actually perform the searches but instead examined whether the studies would be retrieved if using the elements specified in the review as search terms.

We followed the methods used in a recent study that evaluated the retrieval potential of each element of the PICO model used in the fields of upper gastrointestinal/pancreatic diseases and pregnancy/childbirth [26]. This previous study used a sample of Cochrane reviews to determine whether carefully constructed searches for population, intervention, comparison, or outcome elements could retrieve the EMBASE or PubMed records of studies meeting the inclusion criteria of the specific review. The relative recall of each PICO element was determined using the articles judged relevant for inclusion in the reviews [26]. The previous study found high retrieval rates for all PICO elements except the outcome element, supporting the existing recommendation not to search for outcome-related terms. In the present study, we adapted these methods to investigate conceptual frameworks used for qualitative systematic reviews. Specifically, we used 71 qualitative reviews (65 from JBI and 6 from Cochrane) published from 2013 through 2017 that included a total of 927 qualitative studies [9]. From these 71 reviews, we randomly selected reviews to achieve a minimum of a 10% sample of included studies using the true random number generator at Random.org. An overview of the 12 selected reviews is available in Appendix 1. To enable comparisons between databases, only studies included in these reviews that were indexed in both PubMed and CINAHL were included in the present study. Consequently, from a total of 210 studies included in the 12 reviews, we included 101 studies that were indexed in both PubMed and CINAHL.

We focused on PubMed and CINAHL, as they are commonly recommended among health sciences researchers searching for qualitative research. Furthermore, in a study of the coverage of qualitative health research in bibliographic databases, a combination of PubMed and CINAHL retrieved 82% of publications [9]. Although the previous study also found that a combination of Scopus and CINAHL or Scopus and Proquest Dissertations and Theses had higher retrieval rates (about 89%), Scopus does not have a controlled vocabulary of its own, whereas the aim of the present study was to investigate whether elements of conceptual frameworks are present in publication titles, abstracts, or controlled vocabulary.

For each of the 101 included studies, data were recorded using a template containing the following information:

- Bibliographic information about the publications as reported in the review
- Review characteristics (i.e., Cochrane or JBI, review title, presence of conceptual framework elements)
- PubMed bibliographic information (i.e., title, PMID, abstract and author keywords, publication type, MeSH terms)
- CINAHL bibliographic information (i.e., title, publication accession number, abstract and author keywords, subject headings, drug index terms, and other index terms)

We determined relative recall based on whether an included study could be retrieved if a particular element from the conceptual framework was used in the search strategy. A particular study was considered retrievable if at least one of the search terms for an element in the conceptual framework was identified in the bibliographic record, which we noted in the data as “1.” If we were not able to identify any of the search terms for an element, we noted this as “0” in the data.

To describe the data collection process in further detail, a review in this study that presents a synthesis of qualitative evidence of parents' and informal caregivers' views and experiences of communication about routine childhood vaccination can serve as an example [28]. The following elements are described in the review: patient/population, intervention/phenomenon of interest, second intervention/phenomenon of interest, and research type. One of the studies included in this review is on parents' choices and rationales for alternative vaccination schedules [29]. In the PubMed and CINAHL records for this study, the interventions are not described in the abstract or subject headings, meaning that a thorough search using the intervention element would not retrieve this study, whereas it would be retrievable using patient/population and research type elements.

Data were extracted by an experienced information specialist with a background in information science (Tove Faber Frandsen), and extracted data were validated by the two other authors: an experienced information specialist with a background in biomedicine (Mette Brandt Eriksen) and a nurse researcher with extensive qualitative research experience (Christina Louise Lindhardt). Any conflicts were resolved by consensus. Data were extracted from February to May 2020, and validation took place from May to September 2020. The data extraction form is available in Appendix 2.

## RESULTS

The reviews included in this study used varying conceptual frameworks. All reviews included patient/population, intervention/phenomenon of interest, and research type. A few reviews included two different elements describing intervention/phenomenon of interest. Most reviews included either outcome or context but not both elements.

Table 1 provides an overview of the relative recall for each element in PubMed and CINAHL using either text words or subject headings. Relative recall was high for some elements. Relative recall for patient/population was high due to the frequent presence of text words describing this element; it was 97.0% for text words in both databases but was lower for subject headings: 88.1% and 84.2% in PubMed and CINAHL, respectively. Relative recall for research type was 86.1% and 85.1% for text words in PubMed and CINAHL, respectively, but differed dramatically between databases for subject headings: 51.5% in PubMed and 94.1% in CINAHL. Relative recall for the remaining elements were all below 70%. Some reviews included a second element describing intervention/phenomenon of interest, and relative recall in these cases was very low (14% for text words and 7% for subject headings in both databases).

**Table 1:** Relative recall of conceptual framework elements using text words or subject headings in PubMed and CINAHL

Conceptual framework element	PubMed Text words (%)	CINAHL Text words (%)	PubMed MeSH terms (%)	CINAHL Subject headings (%)
Patient/population	97.0	97.0	88.1	84.2
Intervention/phenomenon of interest	65.3	64.4	40.6	38.6
Second intervention/phenomenon of interest	14.3	14.3	7.1	7.1
Research type	86.1	85.1	51.5	94.1
Outcome	60.7	58.9	35.7	39.3
Context	54.8	51.6	48.4	45.2

Table 1 shows differences between databases as well as between the use of text words or subject headings. However, current recommendations for systematic searches stress the importance of using several databases to retrieve relevant literature as well as using both text words and subject headings [30, 31]. Thus, Table 2 provides an overview of the relative recall for each element in PubMed and CINAHL using text words and subject headings together as well as for both databases combined.

**Table 2:** Relative recall of conceptual framework elements using both text words and subject headings in PubMed, CINAHL, and both databases combined

Conceptual framework element	PubMed Text words + MeSH terms (%)	CINAHL Text words + subject headings (%)	PubMed and CINAHL combined Text words + subject headings (%)
Patient/population	99.0	98.0	99.0
Intervention/phenomenon of interest	72.3	70.3	75.3
Second intervention/phenomenon of interest	14.3	14.3	16.7
Research type	89.1	97.0	97.0
Outcome	75.0	73.2	78.6
Context	61.3	54.8	61.3

Relative recall for patient/population was high in both databases (99% in PubMed and 98% in CINAHL); using both databases, 99% of studies would be retrieved. Relative recall for research type was also high: 89.1% in PubMed, 97% in CINAHL, and 97% for both databases. Using text words and subject headings together, relative recall for intervention/phenomenon of interest and outcome increased to 75.3% and 78.6% for both databases respectively. Relative recall for context and the second intervention/phenomenon of interest was lower.

Elements of conceptual frameworks are typically used in combination, and adding more elements to a search strategy can lead to lower retrieval. Table 3 provides an overview of retrieval associated with various combinations of elements. Patient/population and research type, which were the elements with the highest relative recall, provided the highest recall when combined. However, adding intervention/phenomenon of interest, context, or outcome considerably reduced recall.

**Table 3:** Recall of combinations of conceptual framework elements using both text words and subject headings in PubMed and CINAHL combined

Conceptual framework element combinations	PubMed and CINAHL combined Text words + subject headings (%)
Patient/population, research type	96.0
Patient/population, research type, outcome	76.8
Patient/population, research type, intervention/phenomenon of interest	73.3
Patient/population, research type, context	58.1

In summary, when searching PubMed and CINAHL, relative recall was high for patient/population and research type elements. Somewhat lower relative recall, but still above 75%, was found for intervention/phenomenon of interest and outcome elements. Finally, relative recall was lowest for context and the second intervention/phenomenon of interest element. When combining elements, the highest recall was found for a combination of patient/population and research type.

## DISCUSSION

The results of this study show that typical elements of conceptual frameworks appearing in the titles, abstracts, or controlled vocabulary of qualitative systematic reviews are patient/population, intervention/phenomenon of interest, and research type. Other elements used are a second intervention/phenomenon of interest, outcome, and context. The latter two were not used in combination.

The relative recall of these elements varied considerably. Assuming that existing guidelines are followed and thus both PubMed and CINAHL are searched using text words and subject headings, we found high relative recall for patient/population and research type (99% and 97%, respectively). The relative recall of intervention/phenomenon of interest was 74.3%, which was similar to that of outcome (78.6%). Finally, the relative recall for context was relatively low (61.3%).

A recent study that examined the effect of using the PICO model to develop search strategies for quantitative reviews showed that the relative recall of PICO elements was high (100% for population, 100% for intervention, and no less than 90% for comparison and outcome) [26]. Considering qualitative reviews, however, we found that the relative recall of elements in conceptualization frameworks was much lower. The findings of this study suggest that searching the literature for a qualitative review requires careful planning and maybe even the use of several strategies to compensate for the lower recall of many of the elements of conceptual frameworks. If possible, patient/population and research type should be included in the search strategy, although the latter should only be included if the search is also performed in CINAHL. The remaining elements should only be used in the search strategy with care. As the recall associated with some elements is relatively low, search strategies for qualitative literature using conceptualization tools for PubMed and CINAHL should be supplemented with other strategies for finding relevant literature.

Although it indexes a relatively small number of journals [32], CINAHL is often recommended for finding qualitative studies. CINAHL can yield a high number of relevant studies, including those not available in other databases [9, 33]. Based on the findings of the present study, CINAHL should be considered for qualitative systematic reviews due to its high-quality indexing of research types. Furthermore, the challenges of finding relevant literature for qualitative systematic reviews are well known [3] and include a lack of indexing of qualitative methods [14]. However, the present findings indicate that the relative recall of research type is 97% when searching CINAHL.

Although conceptual frameworks can be used to plan the search strategy for a systematic review, some of their elements may not provide a satisfactory level of recall. When planning qualitative reviews, reviewers should be aware of low relative recall when searching for interventions/phenomenons of interest, outcome, and context. Higher relative recall is found when searching for patients/population and research type, although no elements provide 100% recall. Consequently, additional approaches should be considered when locating relevant qualitative studies. In the process of developing search strategies, it should also be taken into consideration that the relative recall of elements depends on the indexing policies of the databases searched.

Potential limitations of this study must also be considered. First, this is a case study, which has limited generalizability. Few empirical studies of search processes for finding qualitative studies exist [14, 34]. Thus, this study aimed to substantiate the assumption that when using a conceptual framework for qualitative reviews, some of its elements will result in higher recall than other elements. More research is needed to substantiate these findings. Second, we defined elements according to a selection of qualitative reviews published by Cochrane and JBI. The studies included in these reviews may not necessarily define the elements of the conceptualization framework identically to those in the review. For example, an included study could define the population more narrowly than the review, which would have an effect on recall. In this case, we chose to take the perspective of a systematic reviewer who develops a search strategy using elements from the conceptualization framework. Consequently, we used reviews' definitions of conceptual framework elements. Furthermore, we used reviews from Cochrane and JBI because they follow a set of strictly established guidelines and undergo rigorous scrutiny [35–37], whereas other reviews do not necessarily have the same characteristics. Third, we did not address subfield differences. Qualitative synthesis approaches include a wide range of methodologies including meta-ethnographies, thematic analyses, and narrative syntheses [38], for which elements of conceptual frameworks may have different relative recall. Fourth, we did not reproduce search strategies in the qualitative reviews and therefore do not know how they located the included studies. Hand-searching, snowballing, searching additional databases, and using other strategies may compensate for low database retrieval. Finally, we made subjective assessments of synonyms for text words and subjects headings. However, as qualitative concepts are complex, the use of varying synonyms and definitions can make it difficult to definitively determine relative recall. We tried to take this into account by validating the extracted data twice, which confirmed that although the assessments were subjective, the process was reproducible.

In conclusion, the results of this case study show that the relative recall of conceptual framework elements varies considerably, suggesting a need to consider existing recommendations regarding the development of search strategies for qualitative systematic reviews. Taking the limitations of this study into account, it appears that the elements of conceptual frameworks developed for qualitative reviews have lower relative recall than the original PICO elements. This suggests that qualitative systematic reviewers must plan searches carefully, as the use of some elements can lower recall considerably. Although patient/population and research type had high recall, other elements should be used with great care or perhaps in different combinations with patient/population and research type. Furthermore, this study confirms the previously noted success of using CINAHL to search for qualitative literature using a conceptual framework.

## Data Availability

All data associated with this article are available in the Open Science Framework: http://doi.org/10.5281/zenodo.4548725.

## SUPPLEMENTAL FILES

**Appendix 1:** Qualitative systematic reviews used as golden standard in this study

**Appendix 2:** Data extraction template
