# Supplementary material for: Performance of conceptual framework elements for the retrieval of qualitative health literature: a case study
Source: J Med Libr Assoc. 2021 Jul 1;109(3):388–94. doi: 10.5195/jmla.2021.1150 (PMC8485961; doi:10.5195/jmla.2021.1150)
Supplement: Supplementary file 1 — Appendix 1: Qualitative systematic reviews used as golden standard in this study [file jmla-109-3-388-s01.docx]

Appendix 1

Qualitative systematic reviews used as golden standard in this study:

- Ames HM, Glenton C, Lewin S. Parents' and informal caregivers' views and experiences of communication about routine childhood vaccination: a synthesis of qualitative evidence. [Cochrane](file:///C:\Users\charlene.dundek\Downloads\Cochrane) Database Syst Rev. 2017 Feb;2(2):CD011787. DOI: https://doi.org/10.1002/14651858.CD011787.pub2.
- Burns T, Fernandez R, Stephens M. The experiences of adults who are on dialysis and waiting for a renal transplant from a deceased donor: a systematic review. JBI Database System Rev Implement Rep. 2015 Mar;13(2), 169–211. DOI: https://doi.org/10.11124/jbisrir-2015-1973.
- Butcher DL, MacKinnon K, Bruce A, Gordon C, Koning C. Experiences of pre-licensure or pre-registration health professional students and their educators in working with intra-professional teams: a qualitative systematic review. JBI Database System Rev Implement Rep. 2017 Apr;15(4): 1011–1056. DOI: https://doi.org/10.11124/JBISRIR-2016-003009.
- Cowley A, Evans C, Bath-Hextall F, Cooper J. Patient, nursing and medical staff experiences and perceptions of the care of people with palliative esophagogastric cancer: a systematic review of the qualitative evidence. JBI Database System Rev Implement Rep. 2016 Oct;14(10): 134–166. DOI: https://doi.org/10.11124/JBISRIR-2016-003168.
- Davis K, White S, Stephenson M. The influence of workplace culture on nurses' learning experiences: a systematic review of qualitative evidence. JBI Database System Rev Implement Rep. 2016 Jun;14(6):274–346. DOI: https://doi.org/10.11124/JBISRIR-2016-002219.
- Dawson S, Jordan Z, Attard M. Carers' experiences of seeking help for relatives with first-episode psychosis: a systematic review of qualitative evidence. JBI Database System Rev Implement Rep. 2013 Nov;11(11):183–361. DOI: https://doi.org/10.11124/jbisrir-2013-1133.
- Glenton C, Colvin CJ, Carlsen B, Swartz A, Lewin S, Noyes J, Rashidian A. Barriers and facilitators to the implementation of lay health worker programmes to improve access to maternal and child health: qualitative evidence synthesis. Cochrane Database Syst Rev. 2013 Oct;2013(10):CD010414. DOI: https://doi.org/10.1002/14651858.CD010414.pub2.
- Jordan J, Rose L, Dainty KN, Noyes J, Blackwood B. Factors that impact on the use of mechanical ventilation weaning protocols in critically ill adults and children: a qualitative evidence-synthesis. Cochrane Database Syst Rev. 2016 Oct;10(10):CD011812. DOI: https://10.1002/14651858.CD011812.pub2.
- Lins S, Hayder-Beichel D, Rücker G, Motschall E, Antes G, Meyer G, Langer G. Efficacy and experiences of telephone counselling for informal carers of people with dementia. Cochrane Database Syst Rev. 2014 Sep:2014(9):CD009126. DOI: https://doi.org/10.1002/14651858.CD009126.pub2.
- Munabi-Babigumira S, Glenton C, Lewin S, Fretheim A, Nabudere H. Factors that influence the provision of intrapartum and postnatal care by skilled birth attendants in low- and middle-income countries: a qualitative evidence synthesis. Cochrane Database Syst Rev. 2017 Nov;11(11):CD011558. DOI: https://10.1002/14651858.CD011558.pub2.
- Tanywe A, Matchawe C, Fernandez R. The experiences of people living with epilepsy in developing countries: a systematic review of qualitative evidence. JBI Database System Rev Implement Rep. 2016 May;14(5):136–192. DOI: https://doi.org/10.11124/JBISRIR-2016-002182.
- Watts R, Huaqiong Z, Shields L, Taylor M, Munns A, Ngune I. Family-centered care for hospitalized children aged 0-12 years: a systematic review of qualitative studies. JBI Database System Rev Implement Rep. 2014;12(7):204–283. https://doi.org/10.11124/jbisrir-2014-1683.
