# Supplementary material for: Performance of conceptual framework elements for the retrieval of qualitative health literature: a case study
Source: J Med Libr Assoc. 2021 Jul 1;109(3):388–94. doi: 10.5195/jmla.2021.1150 (PMC8485961; doi:10.5195/jmla.2021.1150)
Supplement: Supplementary file 2 — Appendix 2: Data extraction template [file jmla-109-3-388-s02.docx]

| **Publication nr.** |  |
| --- | --- |
| **Publication reference** | |
|  | |

# **Publication characteristics:**

# **Review characteristics**

| **Cochrane or Joanna Briggs** | |  |
| --- | --- | --- |
| **Review** | |  |
| **Review title** |  | |

# **Reported findings**

| **Characteristic** | **PubMed**  **Retrievable in**  **TI-AB-KEY (0 = no, 1 = yes)** | **PubMed**  **Retrievable in MeSH**  **(0 = no, 1 = yes)** | **Cinahl**  **Retrievable in TI-AB-KEY**  **(0 = no, 1 = yes)** | **Cinahl**  **Retrievable in subject heading**  **(0 = no, 1 = yes)** |
| --- | --- | --- | --- | --- |
| P: patient / population | 0/1 | 0/1 | 0/1 | 0/1 |
| I: Intervention/ phenomenon of interest | 0/1 | 0/1 | 0/1 | 0/1 |
| R: Research types | 0/1 | 0/1 | 0/1 | 0/1 |
| O: outcomes | 0/1 | 0/1 | 0/1 | 0/1 |

# **Data extraction and validation information**

| **Date (initial data extraction)** |  | **Date (validation)** |  |
| --- | --- | --- | --- |

# **Review characteristics**

| **P: patient / population** |  |
| --- | --- |
| I: Intervention/ phenomenon of interest |  |
| R: research types |  |
| O: outcomes |  |

# **PubMed bibliographic information**

| **Title** |
| --- |
|  |
| **PMID** |
|  |
| **Abstract and author keywords** |
|  |
| **Publication types, MeSH terms, Substances** |
|  |

# **Cinahl bibliographic information**

| **Title** |
| --- |
|  |
| **Publication accession no.** |
|  |
| **Abstract and author keywords** |
|  |
| **Subject headings, drug index terms & other index terms** |
|  |
